# Physical activity counselling among GPs: a qualitative study from Thailand

**DOI:** 10.1186/s12875-019-0968-x

**Published:** 2019-05-29

**Authors:** Apichai Wattanapisit, Sanhapan Thanamee, Sunton Wongsiri

**Affiliations:** 1 School of Medicine, Walailak University, Thasala, Nakhon Si Thammarat, 80161 Thailand; 2 Walailak University Hospital, Thasala, Nakhon Si Thammarat, 80161 Thailand; 3 Thasala Hospital, Thasala, Nakhon Si Thammarat, 80160 Thailand; 4 Department of Orthopedic Surgery and Physical Medicine, Faculty of Medicine, Prince of Songkla University, Hat Yai, Songkhla, 90110 Thailand

**Keywords:** Counselling, General practitioner, Physical activity, Physician, Primary care

## Abstract

**Background:**

Physical activity (PA) counselling is an intervention to promote PA among patients in primary care, yet it remains limited in this clinical setting. This study aimed to explore PA counselling practices among general practitioners (GPs), as well as barriers to and recommendations for improving PA counselling.

**Methods:**

This qualitative study employed a descriptive approach. Data were collected by in-depth interviews and analysed by thematic analysis. The study was conducted in district hospitals in Nakhon Si Thammarat, Thailand, from February 2017 to February 2018. The study participants were GPs who worked at district hospitals.

**Results:**

Seventeen GPs (6 males and 11 females, mean age 29.8 ± 3.4 years) from 6 district hospitals were interviewed. Their clinical experience ranged from 2 to 12 years (mean 4.7 ± 2.9). The GPs saw 30–80 outpatients/day (mean 56.2 ± 14.1) and spent 1–8 min (mean 3.8 ± 1.8) with each patient. They emphasised PA in patients with poorly controlled non-communicable diseases (NCDs) using the word ‘exercise’ in the Thai language as well as discussing time and frequency of exercise. PA was considered a non-pharmacological treatment in the management of NCDs, which improved patient health and quality of life. Barriers to PA counselling among GPs included time constraints, insufficient knowledge, and lack of opportunities to follow-up previously counselled patients. GPs suggested that training in PA counselling was required.

**Conclusions:**

GPs concurred that PA counselling is essential in the treatment of NCDs. Physicians’ recommendations to improve the quality of PA counselling in primary care include 3 Ts: training in PA counselling, tools for prescribing PA, and teams of healthcare professionals. Implementing use of written PA prescriptions may encourage patients to increase PA. Multidisciplinary teams should be developed to support PA counselling in the clinical setting. Further studies should focus on appropriate techniques to implement PA counselling and overcome existing barriers.

## Background

The World Health Organization (WHO) reports that non-communicable diseases (NCDs) are responsible for 63% of all deaths worldwide [1]. Around three-quarters of heart disease, stroke and type 2 diabetes, as well as 40% of cancer could be prevented by eliminating major risk factors, including physical inactivity, unhealthy diet, tobacco use and alcohol abuse [2]. Specifically, physical inactivity causes more than a million deaths each year and contributes to billions of dollars in direct and indirect economic losses [3, 4]. Accordingly, the WHO prioritises increasing physical activity (PA) among the world population as a global target [5]. Global and national recommendations regarding PA are widely provided to promote and facilitate exercise guidance and healthier lifestyles [6, 7].

PA counselling in clinical settings is a promising intervention to promote PA since this approach increases exercise levels, physical functions and quality of life [8–11]. A systematic review estimates that PA promotion in primary care can change 1 out of 12 sedentary patients to become physically active [12]. Compared to smoking cession advice, physicians need to counsel 35–120 smokers to change 1 smoker becomes a non-smoker [13]. Hence, PA promotion is thus an effective approach and remains among the best interventions capable of increasing exercise [14–16]. Moreover, physicians are perceived as powerful motivators armed with credible and reliable information regarding PA [17, 18]. Encounters between physicians and patients provide chances for PA counselling and lifestyle discussions [19, 20]. However, barriers to providing PA counselling in clinical practice, such as time constraints and lack of knowledge or training, remains a challenge [21].

Physicians are expected to possess competence in offering PA counselling while preventing, treating and managing diseases [22]. In Thailand, physician competence in health promotion issues is assessed by criteria set forth by national medical licencing requirements [23]. However, PA counselling is not specified as a compulsory topic in medical education curricula. To the best of our knowledge, PA counselling has not received adequate attention by Thai physicians and researchers. According to the Thai healthcare system, general practitioners (GPs) usually refer to medical graduates or non-specialised physicians. GPs can work in any primary care settings such as, public hospitals, private hospitals, and private clinics. The majority of GPs are mandated to work at general and district hospitals during their first three years after graduation. Then, they have an option to continue the GP status at district hospitals or participate in specialty training. Most district hospitals in Thailand provide hospital-based primary care services, NCD clinics, and inpatient services. Therefore, GPs are expected to run those services. All Thai people are guaranteed access to free healthcare services in the public sectors under the universal health coverage system, including, the Universal Coverage Scheme (75% of the Thai population), the Social Security Scheme (16%), and the Civil Servant Medical Benefit Scheme (9%) [24].

This study therefore aimed to explore PA counselling practices among Thai GPs and barriers to such clinical intervention. This study also obtained practitioners’ viewpoints for improving PA counselling practices in primary care.

## Methods

### Study design and context

This qualitative study employed a descriptive approach to explore a deeper understanding of PA counselling, barriers to such practices, and recommendations to improve counselling in the primary care setting. Semi-structured in-depth interviews (IDIs) were conducted in Nakhon Si Thammarat province, the most populated province in southern Thailand from February 2017 to February 2018. GPs, who worked at district hospitals, were invited to participate in this study.

### Sampling and recruitment

We recruited both male and female GPs with more than 1 year of clinical experience by purposive sampling. We excluded physicians who were certified specialists or in specialist training. We also applied snowball sampling to reach participants with characteristics of interest through their social networks [25]. We made phone calls to eligible GPs or met them at their clinics to ask their verbal permission to participate in the study. Subsequently, we scheduled interviews with participants via telephone and social networks and then conducted all IDIs at their places of work.

### Data collection

A family physician (AW), trained and experienced in qualitative interviewing, conducted all IDIs in Thai. The interviewer provided each participant with an information sheet before the interview. Participants were asked to complete a short questionnaire about personal information and characteristics of outpatients seen in practice. The interviewer interviewed the participants using the interview guide (Table 1) with probing questions to explore the meaning of answers. IDIs were recorded with a digital audio recorder with permission of participants. Each IDI took 30–60 min. We did not identify the initial target sample size. The number of IDIs depended on data saturation, which new data repeat what was revealed in previous data [26].

**Table 1:** Interview guide

Questions	
• How do you provide physical activity counselling to patients?	
• How often do you counsel patients about physical activity?	
• To what type of patients do you provide physical activity counselling?	
• What do you think about written physical activity prescriptions?	
• What reasons make you provide physical activity counselling?	
• What are barriers to physical activity counselling?	
• What do you recommend to improve physical activity counselling for patients?	

### Data analysis

All audio-recorded files were transcribed verbatim. We managed the transcripts using Microsoft Word (the Office 365 University package, Microsoft Inc., Redmond, WA, USA) and printed documents (hard copies). Participants’ names were coded to ensure confidentiality and anonymity. We analysed data using the deductive thematic approach [27, 28]. First, two researchers (AW and ST), both trained and experienced in qualitative methods, read and reread the first transcript to familiarise themselves with the data obtained. The researchers then independently performed initial coding, where codes were assigned throughout the transcripts based on study objectives. Initial codes were merged to form initial thematic maps. Subsequently, the researchers defined and named final themes. The researchers compared analytical similarities and differences. A third researcher (SW) was involved in consensus to resolve any differences in data analysis. Themes and quotations were translated from Thai to English by one of the researchers (AW) at the time of manuscript writing, and all researchers approved the translation.

### Ethical approval

This study was approved by the Human Research Ethics Committee of Walailak University (protocol number WUEC-16-006-01). All participants voluntarily took part in this study and provided written informed consent.

## Results

**Fig. 1:**
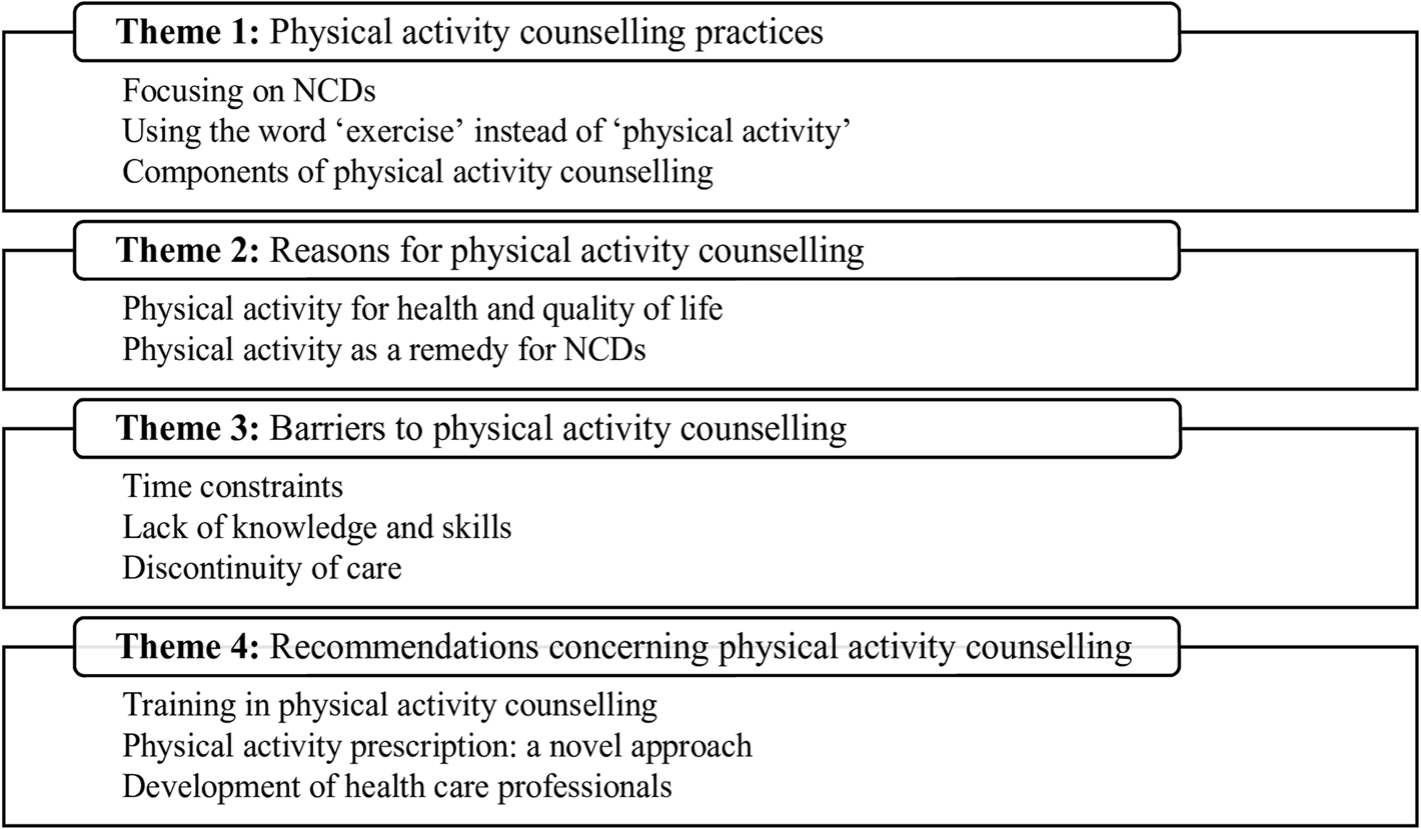
Summary of themes and sub-themes

We conducted 17 IDIs in 6 hospitals and reached the point of data saturation, when there was enough information and no emerging idea was expressed. Eleven participants (64.7%) were female physicians. The mean age of participants was 29.8 ± 3.4 years. Most participants (94.1%, *n* = 16) had less than 10 years of experience in clinical practice as GPs. Participants had 56.2 ± 14.1 outpatients per day and reported an average consultation time of 3.8 ± 1.8 min per patient. Table 2 shows participant codes and characteristics. Findings comprised four emerging themes: (i) PA counselling practices; (ii) reasons for PA counselling; (iii) barriers to PA counselling; and (iv) recommendations concerning PA counselling (Fig. 1).

**Table 2:** Participant codes and characteristics (*n* = 17)

Participant code	Gender	Range of age (year)^a^	Clinical experience (year)	Average number of patients per day	Average time for each consultation (min)
P1	Male	31–35	6	50	5
P2	Female	26–30	2	70	5
P3	Male	31–35	9	60	3
P4	Female	31–35	6	80	3
P5	Male	26–30	6	50	6
P6	Female	26–30	5	50	2
P7	Male	26–30	3	35	3
P8	Female	36–40	12	50	1
P9	Female	26–30	4	60	3
P10	Female	36–40	7	80	5
P11	Female	26–30	3	40	8
P12	Male	26–30	4	50	1
P13	Female	26–30	2	60	2
P14	Female	26–30	4	70	4
P15	Female	26–30	3	30	5
P16	Male	26–30	2	60	4
P17	Female	26–30	2	60	5
Mean (SD)		29.8 (3.4)	4.7 (2.9)	56.2 (14.1)	3.8 (1.8)

^a^Data presented as range of age to avoid the participant’s indirect identifier

### Theme 1: PA counselling practices

#### Focusing on NCDs

According to PA counselling practice, physicians focused on patients with NCDs including hypertension, diabetes mellitus and dyslipidaemia. Patients with poorly controlled conditions were a target group for PA counselling intervention. The rate of PA counselling in patients with NCDs varied among physicians from 10% to almost 100% of cases.

*‘I focus on high-risk patients: obese and overweight. I advise weight reduction, exercise and eating habits. I often talk with obese, chronically ill and other NCD (diabetes, hypertension, and hyperlipidaemia) patients.’*

*P1, 6-year-experienced, 50 patients/day*

*‘Look at blood pressure! If the blood pressure is not good or the sugar level is poorly controlled, I will ask about diet control and exercise. For well controlled patients, I do not ask (about diet control and exercise) because I am in a hurry.’*

*P15, 3-year-experienced, 30 patients/day*

Musculoskeletal disorders were the second most common focus; physicians advised exercise for patients with musculoskeletal disorders.

*‘I talk to patients with knee osteoarthritis, muscle strain or postural imbalances due to their occupations. I will advise to exercise.’*

*P8, 12-year-experienced, 50 patients/day*

#### Using the word ‘exercise’ instead of ‘physical activity’

Physicians used the word ‘exercise’ to communicate with patients. The Thai term for PA was unfamiliar to many patients. Physicians emphasised that occupational PA could not substitute for leisure or recreational PA.

*‘I use the word “exercise” to communicate with patients. It is easier to understand. I ask patients about their exercise or activities at home.’*

*P5, 6-year-experienced, 50 patients/day*

*‘Most patients think exercise and (outdoor) work are the same. I explain that they are different. Even if it (outdoor work) tires you, it is not comparable (with exercise). Exercise can stimulate neurotransmitters. It is absolutely different. I tell patients about this.’*

*P11, 3-year-experienced, 40 patients/day*

#### Components of PA counselling

GPs counselled patients to participate in PA according to the FITT mnemonic (frequency, intensity, time, and type). Time (min/day) and frequency (times/week) of PA were essential information shared with patients. Sometimes, physicians recommended a specific type of PA to patients.

*‘Walk or jog around your neighbourhoods for 30 minutes at least 3 times a week’*

*P12, 4-year-experienced, 40 patients/day*

However, the intensity of PA was rarely discussed with patients. When physicians did inform about intensity, they asked patients to palpate their pulses, feel their heartbeats or otherwise detect their exhaustion.

*‘It is hard to communicate the term “moderate-intensity” to patients. I tell them to feel tired or sweat. Just 30 minutes of exercise is enough; do not be extremely exhausted.’*

*P12, 4-year-experienced, 40 patients/day*

### Theme 2: reasons for PA counselling

#### PA for health and quality of life

Physicians could motivate patients to exercise by providing credible information. If patients were physically active, their health and quality of life would be affected for the better.

*‘There is no financial cost. If patients simply increase their physical activity, it will be beneficial to them. We do not have to invest financially. On the contrary, a drug costs money. It is a good reason to tell patients about the benefits of exercise or physical activity.’*

*P11, 3-year-experienced, 40 patients/day*

#### PA as a remedy for NCDs

Physicians counselled patients to increase PA, apart from prescribing medications and advising diet control, when seeing patients with NCDs.

*‘Treatment does not only involve using drugs. There is a need to collaborate between doctors and patients. As a doctor, I prescribe drugs for my patients and they take them home. They continue to depend on pharmacotherapy. Unless they control their eating habits and exercise, their health will not improve. From my experience, patients who take prescribed drugs while also eating healthily and exercising frequently will experience positive outcomes.’*

*P4, 6-year-experienced, 80 patients/day*

### Theme 3: barriers to PA counselling

#### Time constraints

Time constraints hinder PA counselling due to the large number of patients seen by physicians.

*‘The limitation is time. We have to accept that we have only 1-2 minutes for each patient.’*

*P8, 12-year-experienced, 50 patients/day*

#### Lack of knowledge and skills

Physicians had insufficient knowledge regarding general PA guidelines as well as those for people with diseases. Moreover, communication skills for PA counselling were also insufficient.

*‘If doctors have good communication skills, it will lead to good results. It is not simply telling patients to “go exercise”.’*

*P2, 2-year-experienced, 70 patients/day*

*‘It is about knowledge. I am not sure whether I can choose an appropriate exercise regimen for the patient with a particular disease.’*

*P11, 3-year-experienced, 40 patients/day*

#### Discontinuity of care

Physicians could not follow PA patterns of many patients. Follow-ups were arranged randomly. Therefore, patients did not see a regular physician.

*‘Actually, patients want to see a regular doctor for the continuity of care. Our system does not support this. Each doctor has his own schedule. I cannot guarantee that a patient will see the same doctor every time.’*

*P14, 4-year-experienced, 70 patients/day*

### Theme 4: recommendations concerning PA counselling

#### Training in PA counselling

Training was intended to improve physician practices. Training in PA counselling should be included in medical education curricula or continuing professional development through meetings, knowledge-sharing activities or workshops.

*‘It needs time to change; perhaps 5, 10 or 20 years. Maybe, it should be started from medical school. If medical students see their seniors prescribing exercise, it can be an “organisational culture”. Medical students will learn and follow this pattern and practice. If we want to change, we should begin from medical schools.’*

*P1, 6-year-experienced, 50 patients/day*

*‘For practitioners, there is a need to improve knowledge. Therefore, training can be a half-day or one-day course, meeting or conference.’*

*P5, 6-year-experienced, 50 patients/day*

#### PA prescription: a novel approach

No GPs included in our study had ever heard about a written prescription ordering PA. Such an intervention could be a promising method to improve the effectiveness of PA counselling. However, PA prescriptions may encounter limitations, such as low literacy levels and communication problems, especially in the Thai context.

*‘Physical activity prescriptions have both advantages and disadvantages. It is good to overcome some time constraints this way. When patients go home, they can read the prescription. It is, however, a problem that some patients are illiterate. Many senior people in rural areas cannot see text clearly or cannot read. Sometimes, they do not understand what the doctor writes or they are not able to follow the prescription. I think this is a major drawback.’*

*P16, 2-year-experienced, 60 patients/day*

#### Development of healthcare professionals

Healthcare providers including nurses, physiotherapists and other exercise specialists could support physicians in PA counselling. Accordingly, there was a need to train such staff to be competent in PA counselling as well as being familiar with the practices involved.

*‘We need a multidisciplinary team. It is a priority. We should include one person from each health profession. Then, training should be provided. Health professions should communicate (about physical activity) in the same way.’*

*P3, 9-year-experienced, 60 patients/day*

## Discussion

The GPs emphasised PA in patients with poorly controlled NCDs. They used the word ‘exercise’ in Thai and often discussed time and frequency of exercise. PA was considered a non-pharmacological treatment for NCDs, which improved health and quality of life. Time constraints, insufficient knowledge of PA guidelines and lack of opportunities to follow-up previously seen patients were barriers to PA counselling among GPs. GPs expressed that training in PA counselling was a priority. Written PA prescriptions may be a promising tool in encouraging patients to increase PA levels. In addition, healthcare providers should be trained to support PA counselling in the clinical setting.

GPs in this study expressed that they viewed PA counselling as vital in the management of NCDs. However, counselling might not be enough to effectively improve PA in a poorly controlled group. PA provided several health benefits to patients with symptomatic diseases; moreover, it was important in primary prevention and reduction in disease burden [29]. A worldwide campaign regarding promotion of PA in clinical settings, ‘Exercise is Medicine’, set a goal to make PA assessment and exercise prescription a standard in prevention and treatment of diseases for all patients [30]. Additionally, a systematic review revealed that a majority of primary care providers believed PA promotion was important for all patients [21].

According to our findings, physicians did not use the description of PA to communicate with patients, as it is not a familiar term in the Thai language. Exercise, however, is a common word used interchangeably with PA [31]. Physicians in this study expressed that exercise was a structured and repetitive activity that aimed to improve physical fitness and functions. Hence, occupational PA, characterised by lower exercise intensity and insufficient recovery [32], should not be included when describing PA for clinical treatment purposes. The FITT mnemonic has been commonly used in clinical settings and studies [33–36]. Physicians that participated in this study also applied FITT to counsel about PA. However, the intensity of PA was infrequently discussed with patients. The avoidance of counselling patients for exercise intensity was likely due to time constraints faced by GPs. This gap is very important because the intensity of PA is a main indicator of cardiometabolic benefits [37]. GPs should address this concern and find out an approach to communicate about PA intensity to their patients. One approach is to provide clearer guidance on self-determined PA intensity [38]. The talk test can be a method, which is practical, valid, reliable and inexpensive, to fill such a gap in clinical settings [39–41]. Alternatively, GPs can use the Compendium of Physical Activities, which provides energy expenditure of each PA as a reference to advise patients to participate in any preferred moderate- to vigorous-intensity PA [42].

Physicians in this study had only 3.8 min per consultation. A study conducted in the USA revealed that 75% of primary care providers spend 3–6 min on PA counselling [43]. Considering an average 20-min consultation in America [44], this proportion seems reasonable. However, limited consultation time is a major challenge in Thailand, as a lack of time to counsel patients remains a major problem among primary care providers in different settings [21, 45]. Our findings revealed that insufficient knowledge and lack of skills were also barriers in counselling patients. These findings were consistent with those evaluating primary care providers in other countries [21, 45]. Although training in PA is essential, it may not have received an emphasis in medical school curricula. Previous studies reported that a small amount of time was spent teaching PA in the UK (4.2 h) [46], US (8.1 h) [47] and Australia (12.3 h) [48]. A lack of care continuity led to skipping PA counselling altogether as physicians could not receive feedback from their patients [49].

GPs expressed that training in PA counselling during undergraduate medical education and continuing professional development could be effective. Several studies reported that undergraduate, postgraduate and continuing professional development courses contributed to positive outcomes of knowledge, skills and attitudes regarding PA counselling [50–53]. PA prescription, however, was a new tool of questionable utility to support PA counselling according to study participants. Evidence clearly supported the capacity of PA prescriptions to positively affect patient exercise behaviours and improve metabolic risk factors [54–56]. However, PA prescriptions were considered a questionable modality due to a lack of education in non-pharmaceutical therapy, which reflected that applying PA prescription alone could not ensure its effectiveness [57, 58]. Participating GPs expressed a need to develop multidisciplinary teams to improve the quality of PA counselling. A similar approach, which promoted teamwork among multidisciplinary healthcare professionals, was recommended as an effective method to implement in primary care [59–61].

This qualitative study was conducted in several district hospitals and represented a wide diversity of therapeutic and administrative practices among physicians in different primary care settings. Results included recommendations regarding PA counselling, which were analysed from the perspectives of GPs in real settings. The results could support the understanding of general practice rather than practice in a particular setting. There were two limitations of the study. First, as the participants recruited were early career GPs (aged 26–37 years) from district hospitals, understanding practices of physicians in other age groups remains unclear. Most young GPs are mandated to work at district hospitals prior to specialisation, and we did not contact more experienced physicians for participation in our study. The results might not represent the current practice of mid-career or senior GPs. Second, the qualitative approach we employed may not have yielded results applicable to other physicians in varying practice settings.

### Recommendations

There is a need to improve the quality of PA counselling. As mentioned previously, training in PA is sparse in medical education, especially, PA counselling [62]. Training programmes should be constructed to overcome the important barriers in clinical practice – lack of knowledge and time constraints [63]. Although developing a tool such as a written PA prescription may improve and facilitate PA counselling, GPs may require more time to complete the task. Alternatively, using a PA prescription proforma which used pictures and symbols rather than words may convey the necessary information for illiterate patients or patients with communication difficulties. Therefore, using an effective tool together with a comprehensive model is needed. An example model consists of three steps: PA assessment, counselling and/or PA prescription, and referral to experts [64]. According to this model, GPs have to work with multidisciplinary teams. This approach can help GPs deal with the time constraints in their practices. Maintaining the continuity of care is another element to change patients’ PA behaviours. A systemic structure in healthcare services is required to continue the use of information, the consistent and coherent management, and the ongoing therapeutic relationship [65]. The well-designed system to maintain the continuity of care may compensate the lack of opportunity to see the same physician.

## Conclusions

GPs included in this study expressed that PA counselling is essential in the treatment of NCDs. Counselling should focus on improving exercise in patients with poorly controlled conditions. Improving the quality of PA counselling in primary care needs 3 Ts: training, tools, and teams. GPs recommend initiating widespread training in PA counselling, application of tools for prescribing PA and ample multidisciplinary collaboration across healthcare fields. Implementing use of written PA prescriptions may encourage patients to increase PA. Multidisciplinary teams should be developed to support PA counselling in the clinical setting. Further studies should focus on appropriate techniques to implement PA counselling in clinical settings and overcome existing barriers.

## Data Availability

Data used during the study are available from the corresponding author on reasonable request.

## Abbreviations

FITT: Frequency, intensity, time, and type
GPs: General practitioners
IDIs: In-depth interviews
NCDs: Non-communicable diseases
PA: Physical activity
SD: Standard deviation
UK: United Kingdom
US: United States
USA: United States of America
WHO: World Health Organization
